# Targeting and killing of glioblastoma with activated T cells armed with bispecific antibodies

**DOI:** 10.1186/1471-2407-13-83

**Published:** 2013-02-22

**Authors:** Ian M Zitron, Archana Thakur, Oxana Norkina, Geoffrey R Barger, Lawrence G Lum, Sandeep Mittal

**Affiliations:** 1 Department of Neurosurgery, Wayne State University, Karmanos Cancer Institute, Detroit, MI, USA; 2 Department of Oncology, Wayne State University, Karmanos Cancer Institute, Detroit, MI, USA; 3 Department of Neurology, Wayne State University, Karmanos Cancer Institute, Detroit, MI, USA; 4 Department of Medicine, Wayne State University, and Karmanos Cancer Institute, Detroit, MI, USA; 5 Department of Immunology and Microbiology, Wayne State University, and Karmanos Cancer Institute, Detroit, MI, USA

**Keywords:** High-grade glioma, Adjuvant therapy, Immunotherapy, Activated T cells, Bispecific antibodies

## Abstract

**Background:**

Since most glioblastomas express both wild-type EGFR and EGFRvIII as well as HER2/*neu*, they are excellent targets for activated T cells (ATC) armed with bispecific antibodies (BiAbs) that target EGFR and HER2.

**Methods:**

ATC were generated from PBMC activated for 14 days with anti-CD3 monoclonal antibody in the presence of interleukin-2 and armed with chemically heteroconjugated anti-CD3×anti-HER2/*neu* (HER2Bi) and/or anti-CD3×anti-EGFR (EGFRBi). HER2Bi- and/or EGFRBi-armed ATC were examined for *in vitro* cytotoxicity using MTT and ^51^Cr-release assays against malignant glioma lines (U87MG, U118MG, and U251MG) and primary glioblastoma lines.

**Results:**

EGFRBi-armed ATC killed up to 85% of U87, U118, and U251 targets at effector:target ratios (E:T) ranging from 1:1 to 25:1. Engagement of tumor by EGFRBi-armed ATC induced Th1 and Th2 cytokine secretion by armed ATC. HER2Bi-armed ATC exhibited comparable cytotoxicity against U118 and U251, but did not kill HER2-negative U87 cells. HER2Bi- or EGFRBi-armed ATC exhibited 50—80% cytotoxicity against four primary glioblastoma lines as well as a temozolomide (TMZ)-resistant variant of U251. Both CD133– and CD133+ subpopulations were killed by armed ATC. Targeting both HER2Bi and EGFRBi simultaneously showed enhanced efficacy than arming with a single BiAb. Armed ATC maintained effectiveness after irradiation and in the presence of TMZ at a therapeutic concentration and were capable of killing multiple targets.

**Conclusion:**

High-grade gliomas are suitable for specific targeting by armed ATC. These data, together with additional animal studies, may provide the preclinical support for the use of armed ATC as a valuable addition to current treatment regimens.

## Background

Malignant gliomas, the most lethal brain tumor in adults, account for approximately 13,000 deaths annually in the US [1]. Long-term prognosis for glioblastoma patients remains poor despite surgery and chemoradiotherapy. Major reasons for treatment failure include its highly infiltrative nature and chemoresistance. Given the limitations of aggressive multimodality treatment, targeted cell therapy is an attractive therapeutic alternative.

Despite the paucity of studies, development of cell therapy for glioblastomas has been encouraging. Arming anti-CD3 activated T cells (ATC) with bispecific antibodies (BiAb) that target the T cell receptor on one hand and the tumor-associated antigen on the other can redirect the non-MHC restricted cytotoxicity of ATC to lyse tumors. Arming *ex vivo* expanded T cells with BiAbs may not only improve clinical responses but also minimize toxicity by avoiding the cytokine storm that can occur by systemic infusion of BiAb alone [2]. Arming ATC with HER2Bi or EGFRBi converts every ATC into a specific cytotoxic T cell [3-7]. Our preclinical studies show that armed ATC can target breast [6], prostate [8], ovarian [5] EGFR+ cancers (head & neck, colorectal, pancreatic, lung [4], neuroblastomas [9], and CD20^+^ NHL [7]. ATC armed with HER2Bi were not only able to lyse cancer cells that have high (3+) expression of HER2 but more importantly target and lyse MCF-7 cells that express low or nil HER2 expression [6] Moreover, armed ATC can kill multiple times, secrete cytokines/chemokines and multiply after engaging tumor cells *in vitro*[10]. *In vivo* anti-tumor activity of armed ATC when co-injected with tumor cells to prevent the tumor development or when injected intratumorally into xenograft model of prostate cancer, armed ATC persist in Beige/SCID mice for 91 days in the spleen and bone marrow without interleukin-2 (IL-2) support [8,11]. Intravenous infusions of armed ATC inhibit tumor growth in the xenograft models in colon [4] and ovarian cancer [5]. In our phase I clinical trial involving stage IV breast cancer patients who received activated T cells (ATC) armed with anti-CD3×anti-Her2/*neu* bispecific antibody (HER2Bi), high levels of circulating tumoricidal cytokines and specific cytotoxicity by PBMC were observed [10]. In an earlier trial, using targeted therapy, lymphokine activated killer (LAK) cells armed with chemically heteroconjugated bispecific antibody (anti-CD3MAb x anti-glioma MAb) in 10 patients showed promising clinical results. In 10 patients, 4 patients had regression of tumor and another 4 patients showed histological eradication of remaining tumor cells post surgery with no recurrence in 10–18 months follow-up [12]. ATC armed with HER2Bi and/or anti-CD3×anti-EGFR (EGFRBi) produced by chemical heteroconjugation of anti-CD3 (OKT3) with trastuzumab or cetuximab, respectively, offers a compelling choice for adjuvant immunotherapy following surgery and chemoradiotherapy.

Although immortalized glioma lines can provide useful biologic insights, cell lines from freshly-resected tumors may more accurately represent the behavior of glioma cells *in vivo*. In this study, we first established that primary glioma cells can be killed by armed ATC and then addressed further questions of therapeutic relevance: 1) Does dual targeting with BiAbs by mixing individual populations of EGFRBi- and HER2Bi-armed ATC or arming ATC with both BiAbs simultaneously enhance specific cytotoxicity? 2) Can CD133 enriched, CD133− and unfractionated tumor cells be killed differentially by armed ATC? 3) Will armed ATC eliminate a temozolomide (TMZ) resistant subline of U251MG? 4) Do armed ATC continue to kill after being irradiated and in the presence of TMZ? 5) Does binding of armed ATC to glioma cell lines induce the secretion of cytokines?

## Methods

### Generation and expansion of activated T cells

Anti-CD3-activated ATC were expanded in culture from human peripheral blood mononuclear cells (PBMC) [6]. Briefly, ATC were produced by activating PBMC with 20 ng/ml of soluble anti-CD3 (OKT3, Ortho Pharmaceutical, Raritan, NJ) and expanded in IL-2 (aldesleukin, Prometheus Laboratories Inc., San Diego, CA) (100 IU/ml) in RPMI 1640 medium supplemented with 10% fetal calf serum (FCS), 2 mM L-glutamine, and 1% penicillin-streptomycin for 14 days. After culture, ATC were harvested, washed, counted, and resuspended in RPMI 1640 for immediate use or cryopreserved.

In experiments where ATC were irradiated, cells received a single 2500 cGy dose using a blood bank cell irradiator (Nordion, Ottawa, Canada) to prevent lymphocyte proliferation [10] and determine whether cytotoxic activity of BiAb-armed ATC is radioresistant.

### Bispecific antibodies and arming of ATC

Preparation and characteristics of BiAbs have been described previously [3,6]. HER2Bi was prepared by chemical heteroconjugation of OKT3 and trastuzumab (Herceptin®, Genentech, South San Francisco, CA). EGFRBi was produced by chemical heteroconjugation of OKT3 and cetuximab (Erbitux®, Bristol-Myer Squibb, NY). Anti-CD3×anti-CD20 BiAb (CD20Bi) was made from heteroconjugation of OKT3 and rituximab (Rituxan®, Genentech, South San Francisco, CA) [7]. ATC were armed with BiAbs at 50 ng/10^6^ cells for 1 hour at 4°C, washed, and resuspended in complete RPMI 1640.

### *Ex vivo* primary glioblastoma lines

Tumor tissue was washed with PBS+EDTA (2 mM), chopped into fragments ≤1 mm, and enzymatically digested using Accumax (Innovative Cell Technologies, San Diego, CA). Fragments of undigested tissue were removed by low g sedimentation and cell clumps were removed by tissue sieves. Contaminating erythrocytes were removed by centrifugation over Ficoll-Hypaque. Viable single cells were counted using trypan blue exclusion. Culture of the *ex vivo* adherent differentiated glioma cells was carried out in DMEM-F12 medium (Mediatech, Manassas, VA) supplemented with 10% FCS (Atlanta Biologicals, Atlanta, GA), L-glutamine, and gentamicin (10 μg/ml). Propagation of neurospheres containing cells with stem-like properties was performed in Neurobasal medium (Invitrogen, Carlsbad, CA) containing N-2 and B-27 supplements, human recombinant EGF, and human recombinant basic FGF (each at 20 ng/ml) (PeproTech, Rocky Hill, NJ) [13].

### Long-term glioblastoma lines

Glioma cell lines U87MG, U118MG, and U251MG were also cultured as adherent monolayers in the DMEM-F12-based medium. U87 and U251 cells were grown in 6-well plates in medium supplemented with TMZ over a range of concentrations (10—1000 μM). Medium was changed every 3 days, maintaining the original TMZ concentration. Over 2 weeks, growth of U87MG cells was unaffected, whereas loss of some U251MG cells was recognizable at 10 μM and progressively increased such that a few surviving cells were identified at 333 μM TMZ, but none at 1000 μM. The cells selected in 333 μM TMZ were subsequently propagated in medium containing TMZ (333 μM).

### Antibodies, cell separation, and cellular phenotyping

Monoclonal antibodies (cetuximab, trastuzumab, and rituximab) were labeled with the N-hydroxysuccinimide ester of Alexa Fluor 488 (Invitrogen, Carlsbad, CA). These reagents were used for flow cytometry at 1—10 μg/ml. CD133+ cells were isolated using magnetic bead separation (Miltenyi Biotec, Auburn, CA). Frequency of CD133+ cells was determined by flow cytometry using phycoerythrin- (PE) conjugated monoclonal anti-CD133/2 antibodies (Miltenyi Biotec, Auburn, CA).

### Viability and cytotoxicity assays

#### MTT assay

Target cells (4x10^4^ in a volume of 0.1 ml) were plated in 96-well flat bottom microtiter plates (Corning Inc., Corning, NY) and allowed to adhere. Effector cells were added in a volume of 0.1 ml to achieve the effector:target ratio (E:T) indicated and the plates incubated overnight, allowing 16 hours for killing to occur. All groups were performed in triplicate. Non-adherent effector cells were removed and viability of the remaining target cells determined using MTT assay. Metabolism of MTT to the formazan product is a measure of residual viable cells, in contrast to ^51^Cr release, which is a direct measure of cytotoxicity. We validated the use of the former by confirming a linear relationship between viable cell number and MTT signal and also by performing MTT and ^51^Cr release in parallel and determining the correlation coefficient of the two data sets.

#### ^51^Cr Release assay

This was performed in 96-well flat-bottom microtiter plates [6]. Target cells (4x10^4^ cells/well) were plated and allowed to adhere overnight, labeled with ^51^Cr at 37°C for 4 hours, and then washed *in situ* to remove unincorporated isotope. Subsequently, effectors were added to achieve a given E:T. ^51^Cr release was measured after 18 hours and percent cytotoxicity calculated as follows: (experimental cpm – spontaneous cpm) / (maximum cpm – spontaneous cpm) × 100. Triplicate determinations were performed and the means and standard errors of the triplicates calculated.

### Bio-Plex assay for the measurement of cytokine secretion

Cytokines were quantitated in culture supernatants using 25-plex human cytokine Luminex Assay (Invitrogen, Carlsbad, CA) in the Bio-Plex System (Bio-Rad Lab., Hercules, CA). The multiplex panel includes interleukin-1β (IL-1β), IL-1 receptor antagonist (IL-1Ra), IL-2, IL-2R, IL-4, IL-5, IL-6, IL-7, IL-8, IL-13, IL-17, tumor necrosis factor (TNF)-α, interferon (IFN)-α, IFN-γ, granulocyte-macrophage colony-stimulating factor (GM-CSF), macrophage inhibitory protein (MIP)-1α, MIP-1β, interferon-inducible protein (IP)-10, monokine induced by IFN-γ (MIG), eotaxin, regulated on activation normal T cell expressed and secreted (RANTES), and monocyte chemotactic protein (MCP)-1. The limit of detection for these assays is <10 pg/mL based on detectable signal of >2 fold above background. Cytokine concentration was calculated by the Bio-Plex Manager Software using a standard curve derived from recombinant cytokine standards.

### Statistical analysis

Calculations of means and standard error of the mean (±SEM), non-parametric correlation tests, and 1- and 2-way ANOVA were performed using Prism5 (GraphPad Software, San Diego, CA). All experiments were repeated at least 3 times.

## Results

### Validation of MTT assay

**Figure 1:**
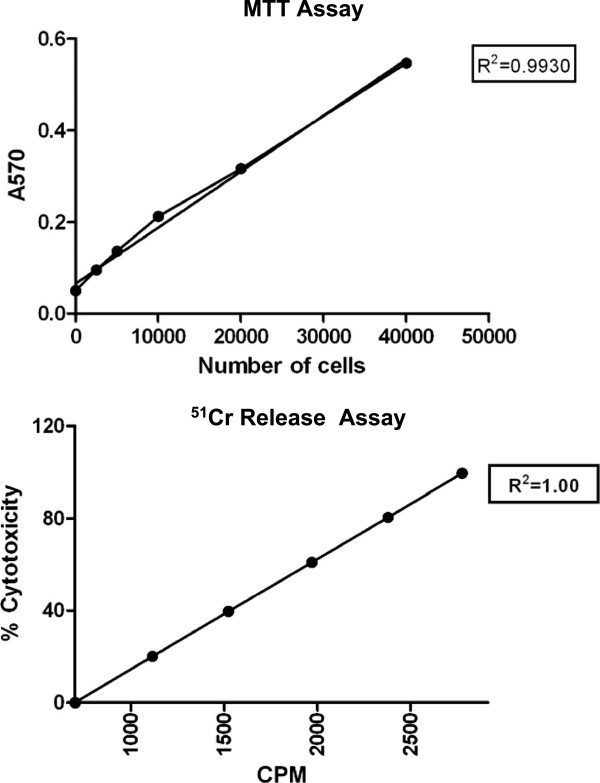
**Linearity of MTT and **^**51**^**Cr responses of glioma lines. Upper panel: **A representative data showing a linear correlation between OD and cell numbers of U251MG glioma cell line. The indicated numbers of U251MG cells were incubated with MTT for 4 hours under standard conditions. The assay was completed by the solubilization of formazan, the A_570_ and A_650 _nm measured, and the difference calculated. **Lower panel:** Shows correlation between counts per minute (CPM) and % specific cytotoxicity using ^51^Cr release assay.

Since our previous work with armed ATC employed the ^51^Cr release assay, we did parallel testing to confirm the relationship between viable cell number and MTT assay signal. We established in the glioma cell lines U251MG (Figure 1) as well as glioma U118MG (data not shown) and breast cancer line SKBR3 (data not shown), that absorbance (A_570_-A_650_) is a linear function (R^2^ = 0.9930) of the cell number up to 4 × 10^4^ cells similar to counts per minute (CPM) as a linear function (R^2^ = 1.0) of cytotoxicity (Figure 1, lower panel). Hence, the MTT assay is a reliable indicator of residual viable cells. Note that when removal of unarmed ATC is incomplete, it may give rise to values >100% in groups that received unarmed ATC or control BiAb-ATC. When ATC were armed with EGFRBiAb or HER2BiAb residual target viability clearly was lower. In several experiments, parallel experiments were performed in replicate 96-well plates tested using MTT and specific cytotoxicity using ^51^Cr release. There was a statistically-significant negative correlation (Spearman r = −0.5804; exact two-tailed p value = 0.0479), validating the MTT assay in this system.

### Optimizing the arming dose of bispecific antibody

**Figure 2:**
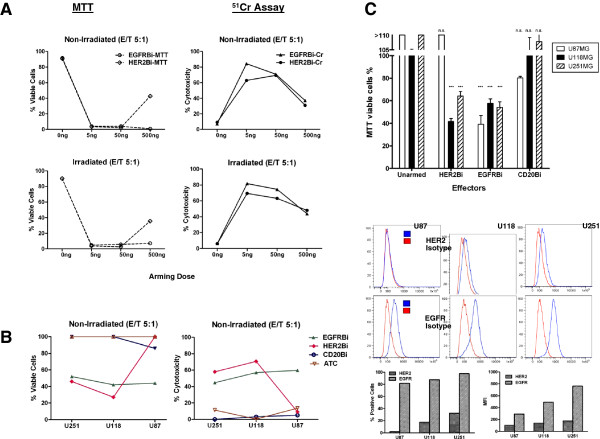
**BiAb-armed ATC specifically kill long term glioma cell lines. 2A**: Non-irradiated or irradiated ATC were armed with the indicated amounts (ng/10^6 ^cells) of either EGFRBi (circles) or HER2Bi (triangles) and used as effector cells at an E:T of 5:1. The figure shows the results from one of two donors, with U118MG cells as targets. Both MTT and ^51^Cr assays were used as readouts. **2B**: Shows the data for U87MG, U118MG, and U251MG cells using non-irradiated unarmed ATC or armed ATC at one arming dose of 50 ng for EGFRBi, HER2Bi and CD20Bi at E/T of 5:1. Both MTT and ^51^Cr assays were used as readouts. **2C**: U87MG, U118MG, and U251MG cells were exposed overnight to the indicated effector cells at an E:T of 5:1 and residual viability determined by MTT assay. U87MG expresses only EGFR whereas U118MG and U251MG express both HER2/*neu *and EGFR. Data were analyzed by 1-way ANOVA: for all 3 glioma lines, overall p < 0.0001; for all 3 lines, unarmed vs. CD20Bi p value is non-significant (p > 0.05) (n.s.). **2C **(lower panel): Shows the expression of EGFR and HER2 (blue) compared to isotype control (red), and plots for percent positive cells and mean fluorescence intensity (MFI). For U87MG, Unarmed vs. HER2Bi, p > 0.05 (n.s.); Unarmed vs. EGFRBi, p < 0.001 (***). For U118MG, Unarmed vs. HER2Bi and Unarmed vs. EGFRBi, p < 0.001 (***). For U251MG, Unarmed vs. HER2Bi and Unarmed vs. EGFRBi, p < 0.001 (***).

The lots of BiAbs were tested on unirradiated and irradiated ATC from two normal donors by arming with doses of 0, 5, 50, and 500 ng of HER2Bi or EGFRBi/10^6^ ATC. A representative data using U118MG targets are shown in Figure 2A, upper and lower panels. Similar arming dose titration results were obtained with U87 and U251 targets (data not shown).

In the MTT assay, ATC armed with EGFRBi killed effectively at all 3 doses, essentially eliminating all U118 target cells, while ATC armed with HER2Bi showed 100% cytotoxicity at 5 ng and 50 ng arming dose, however, cytotoxic activity was reduced at 500 ng dose (Figure 2A, upper panel). Similarly, the ^51^Cr release assay showed higher cytotoxicity at 5 and 50 ng of EGFRBi or HER2Bi/10^6^ ATC with decreasing cytotoxicity as the arming dose was increased to 500 ng of HER2Bi or EGFRBi/10^6^ ATC, which could be due to the receptor saturation induced desensitization of ATC. Similar results were observed with irradiated ATC or aATC at 5, 50 and 500 ng arming doses (Figure 2A, lower panel) and based on these results, we armed ATC at 50 ng of HER2Bi or EGFRBi/10^6^ ATC for subsequent experiments. The data with the three donors in all three cell lines (U87MG, U118MG and U251MG) using unarmed ATC or armed ATC at 50 ng dose are shown in Figure 2B. All experiments were repeated at least three times.

### Specific killing of long-term glioma cell lines by armed ATC

Flow cytometry analysis of U87MG, U118MG, and U251MG confirmed that all 3 lines show high surface expression of EGFR (80-100% cell positivity) while only the latter two expressed low levels of surface HER2/*neu* (U118MG: 17.6% and U251MG: 32.5% positive cells). Figure 2C (lower panel) shows the histograms of EGFR and HER2 expression (blue) compared to isotype control (red), and plots for percent positive cells and mean fluorescence intensity (MFI). The three lines were used as targets for ATC armed with EGFRBi, HER2Bi, and CD20Bi. Figure 2C (upper panel) shows residual viability at E:T = 5:1. Unarmed ATC showed no reduction of tumor cell viability; CD20Bi-armed ATC (irrelevant control) also showed no reduction of tumor cell viability for U118 and U251 cells but showed reduced viability (80%) for U87. CD20Bi-armed ATC mediated reduction in the viability against U87 cells could be due to the nonspecific binding of armed ATC to target cells resulting in effector target interaction induced cytotoxicity. In contrast, all 3 tumor cell lines showed significant losses of viability when exposed to EGFRBi-armed ATC. U118MG and U251MG viabilities were reduced by HER2Bi-armed ATC (middle and right columns), consistent with the surface expression data, whereas HER2Bi-armed ATC (left column) failed to reduce the viability of U87MG cells below 100%. The fetal calf serum used to supplement medium was heat-inactivated and preparation of BiAbs effectively eliminates the complement-fixing properties of the Fc regions, suggesting that aATC mediated cytotoxicity cannot be accounted for complement dependent cytotoxicity.

### Killing of fresh *ex vivo* glioma cells

**Figure 3:**
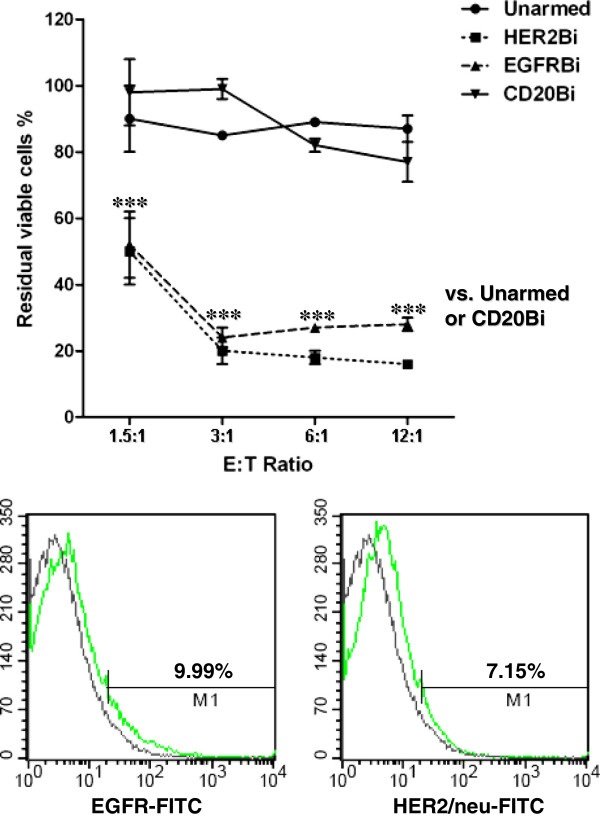
**Tumor cells in primary culture are specifically killed by BiAb-armed ATC. ***Ex vivo *glioma cells expressing both HER2/*neu *and EGFR were incubated overnight with unarmed ATC or ATC armed with HER2Bi, EGFRBi or CD20Bi. The data are pooled from 4 experiments, using 4 different ATC donors and a range of E:T. Residual viabilities (mean ± SEM) based upon MTT assays are shown. The targets are killed by HER2Bi- and EGFRBi-armed ATC, whereas unarmed and CD20Bi-armed ATC fail to kill. Mean of 4 donors (± SEM), when compared with unarmed ATC, the residual viable cells after HER2Bi- or EGFRBi-ATC were statistically significant (p < 0.001, 1-way ANOVA; ***). Arming with CD20BiAb showed no statistically significant difference from unarmed ATC (p > 0.05, 1-way ANOVA; n.s.). Lower panel: Shows the HER2 (7.15%) and EGFR (9.99%) expression as percent positive cells in this *ex vivo *cell line.

Figure 3, upper panel shows susceptibility of one primary glioma cell line derived from resected tissue from a patient with a histologically-confirmed glioblastoma. FACS analysis showed both EGFR (9.99%) and HER2/*neu* (7.15%) expression (Figure 3, bottom panel). The mean residual viabilities (and SEM) for 4 ATC donors are shown when these cells were used as targets, over E:T ratios ranging from 1.5:1 to 12.5:1. The data shown were pooled from 4 experiments. The curves for unarmed ATC and CD20Bi-armed are essentially identical and show residual viabilities fluctuating around 100%. In contrast, both HER2Bi- and EGFRBi-armed ATC reduced viability to 15-20% over a range of E:T from 1.5:1 to 12.5:1. This demonstrates that glioma cells obtained from fresh tumors are appropriate targets and that ATC from different individuals show general agreement in armed ATC function.

### Does the simultaneous Use of Two BiAbs to Arm ATC improve killing glioma targets?

**Figure 4:**
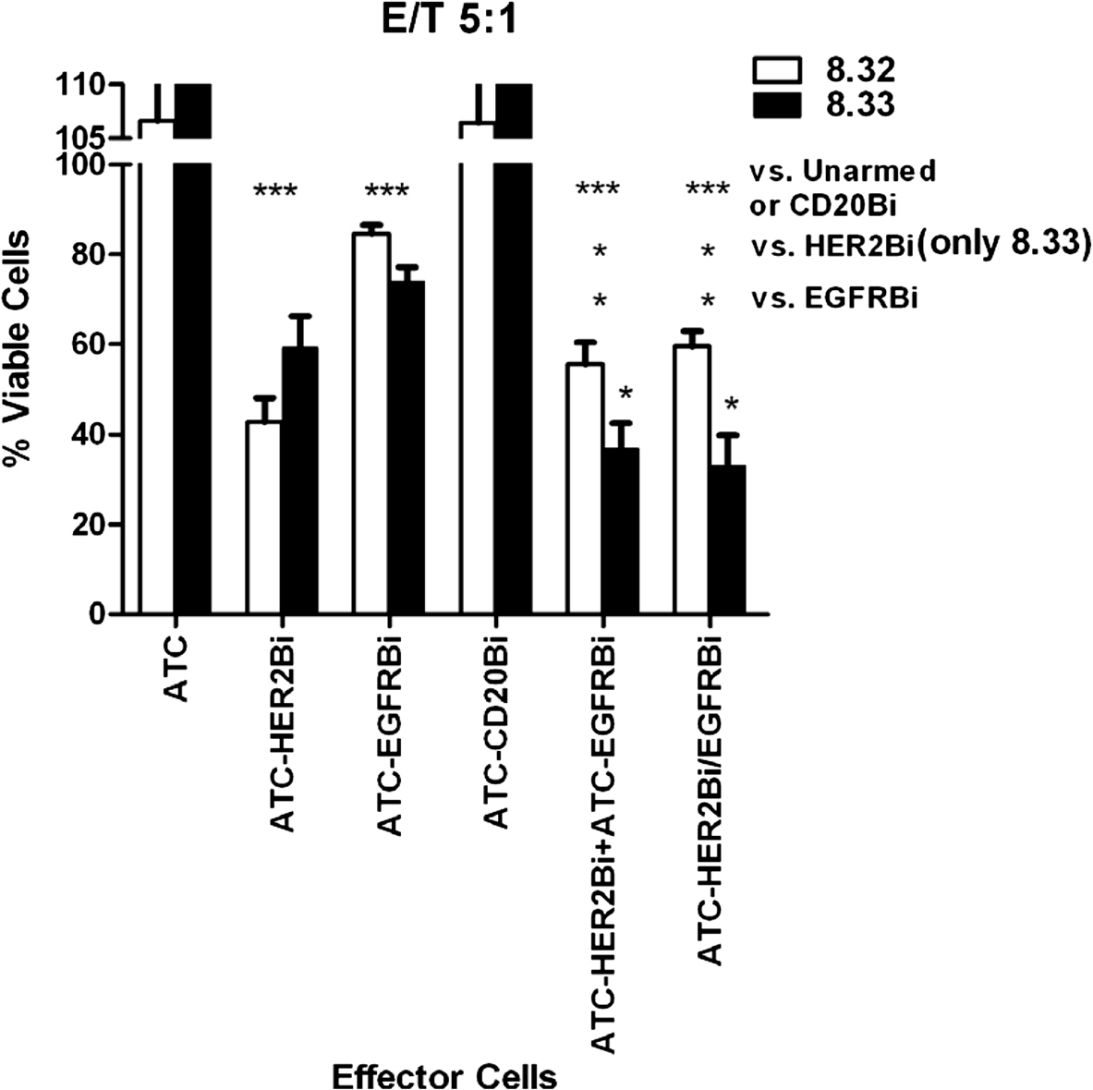
**Targeting ATC to two molecules on the tumor cells’ surfaces enhances killing. **Targets were *ex vivo *cells from two patients with glioblastoma (08–32 and 08–33); both *ex vivo *lines expressed both HER2/*neu *and EGFR. The targets were exposed overnight (E:T = 5:1) to unarmed ATC or ATC populations armed with single BiAb (ATC-HER2Bi, ATC-EGFRBi, ATC-CD20Bi), a mixture of equal numbers of singly-armed HER2Bi- and EGFRBi-ATC (ATC-HER2Bi+ATC-EGFRBi) and a population of ATC simultaneously armed with HER2Bi and EGFRBi (ATC-HER2Bi,EGFRBi). Mean residual viability was determined by MTT assay. For both 08-32 and 08-33 target cells, overall analysis by 1-way ANOVA, p < 0.0001. Unarmed or CD20Bi-armed vs. HER2Bi-, EGFRBi and both doubly armed ATC, p < 0.0001 (***). HER2Bi vs. either doubly-armed ATC, p < 0.05 (only for 8-33). EGFRBi vs. HER2Bi+EGFRBi, p < 0.01 (**). EGFRBi vs. HER2Bi,EGFRBi, p < 0.05 (*). Comparisons between individual effector cells performed using Bonferroni multiple comparison test.

Since there was a spectrum of expression levels of EGFR and HER2/*neu* on the target cells, we investigated whether simultaneous targeting using two BiAbs would improve killing. Two approaches were tested: (i) combining singly-armed populations of ATC effectors; and (ii) arming ATC with EGFRBi and HER2Bi simultaneously. Primary glioma cells from two patients (08–32 and 08–33) were tested with armed ATC from 4 normal donors at an E:T of 5:1 (Figure 4). Both malignant glioma lines were histologically-confirmed specimens. The unarmed and CD20Bi-armed ATC effectors did not exhibit any cytotoxicity. Viabilities were >100% indicating that residual ATC increased the numbers of live cells. The single BiAb-armed ATC showed efficient reduction with HER2Bi-armed ATC apparently being more efficient than EGFRBi-armed cells. Data show evidence for significantly enhanced loss of viable cells due to mixing two effector populations, or to double-arming a single population. One-way ANOVA indicated no statistically significant difference between the HER2Bi-armed ATC and the doubly-armed or mixed single-armed. There was a significant difference when comparing EGFRBi-armed cells with the double- or mixed single-armed ATC, but this reflects the lesser efficiency of the EGFRBi-armed ATC in this assay. Additionally, there is no evidence that mixing or double-arming compromises efficiency, when these two active bispecific antibodies are used. These data show some additive effect at least in one primary glioma cells (8–33) with both BiAbs.

#### Are CD133 enriched cells, CD133− cells and unfractionated tumor cells killed differentially by armed ATC?

**Figure 5:**
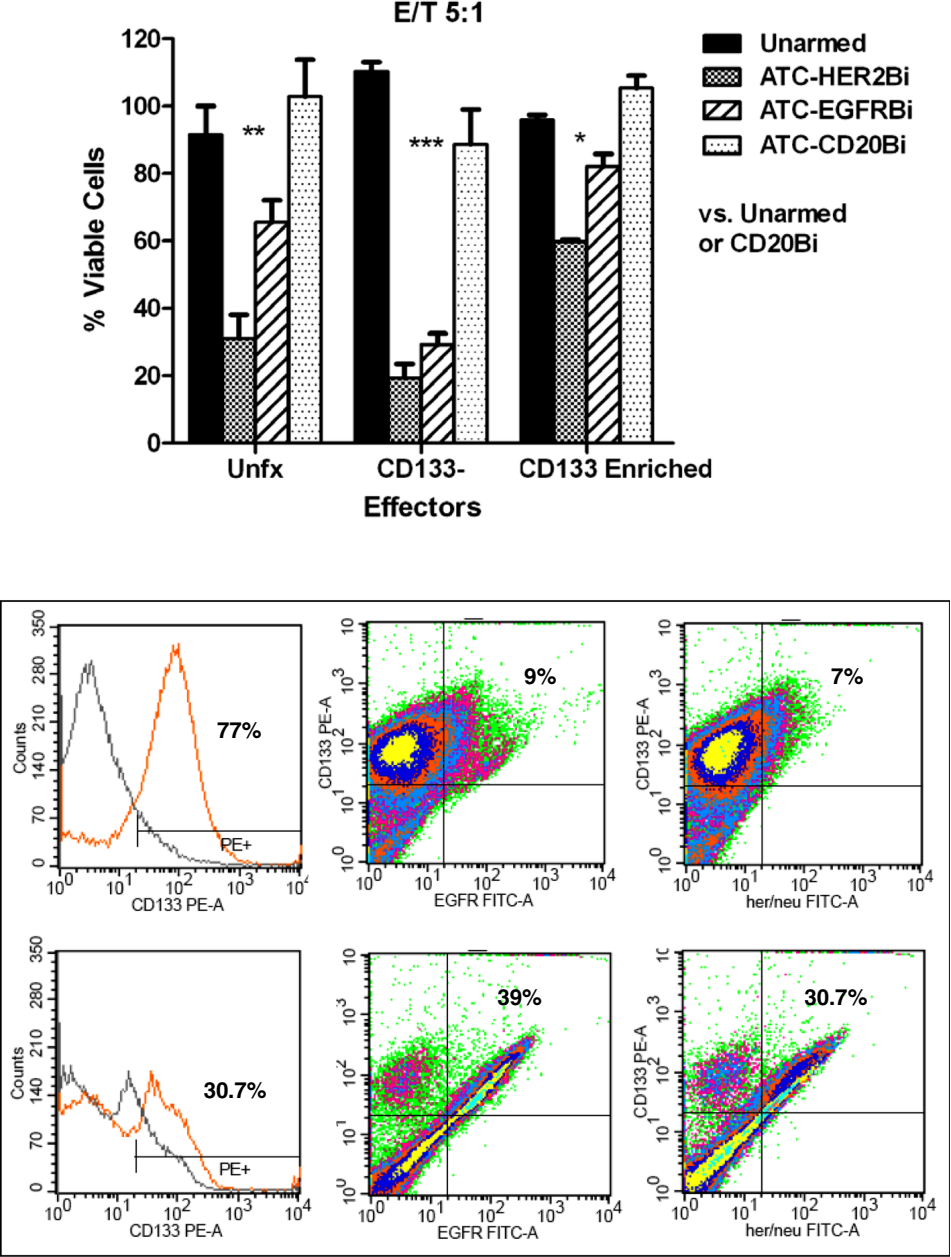
**Both CD133 enriched and CD133– glioma cells are susceptible targets for BiAb-armed ATC. **Cells from an *ex vivo *glioblastoma were separated into CD133– and CD133 enriched populations using magnetic separation (Miltenyi Biotec). The separated populations, stained with a CD133/2-specific MAb are shown in the inset. Unfractionated (Unfx) cells and CD133– and CD133 enriched populations were incubated overnight with unarmed ATC, or ATC armed with HER2Bi or CD20Bi, at an E:T of 3:1. The mean (± SEM) residual viability was determined using the MTT assay. Lower panel: Show the expression of HER2 and EGFR in CD133 enriched population from two *ex vivo *primary cells. Overall analysis by 1-way ANOVA, p < 0.0001. Individual comparison (Tukey-Kramer test): unarmed unfx vs. HER2Bi unfx p < 0.001 (***); unarmed unfx vs. CD20Bi unfx p > 0.05 ; unarmed CD133– vs. HER2Bi CD133–p < 0.001 (***); unarmed CD133– vs. CD20Bi CD133–p < 0.001; unarmed CD133+ vs. HER2Bi CD133+p < 0.05 (*); unarmed CD133+ vs. CD20Bi CD133+p < 0.01.

We tested the susceptibility of unfractionated, CD133 enriched cells and CD133– cells from two *ex vivo* tumor cell lines to killing by armed ATC from 3 normal donors. ATC armed with HER2Bi or EGFRBi showed a significant reduction in viability of all three target populations at 5:1 E:T ratio (Figure 5, upper panel). At the same E:T ratio (5:1), unarmed or CD20-armed ATC showed insignificant effects on viability. Both putative stem cells (CD133 enriched) and the bulk tumor population are killed by armed ATC, at a low E:T (5:1) at which the efficiency of the system is tested critically. Lower panel of Figure 5 shows the expression of HER2 and EGFR in CD133 enriched population from two primary cells.

### Does chemoresistance confer protection against specific cytotoxicity by armed ATC? Are armed ATC effective after having undergone irradiation?

**Figure 6:**
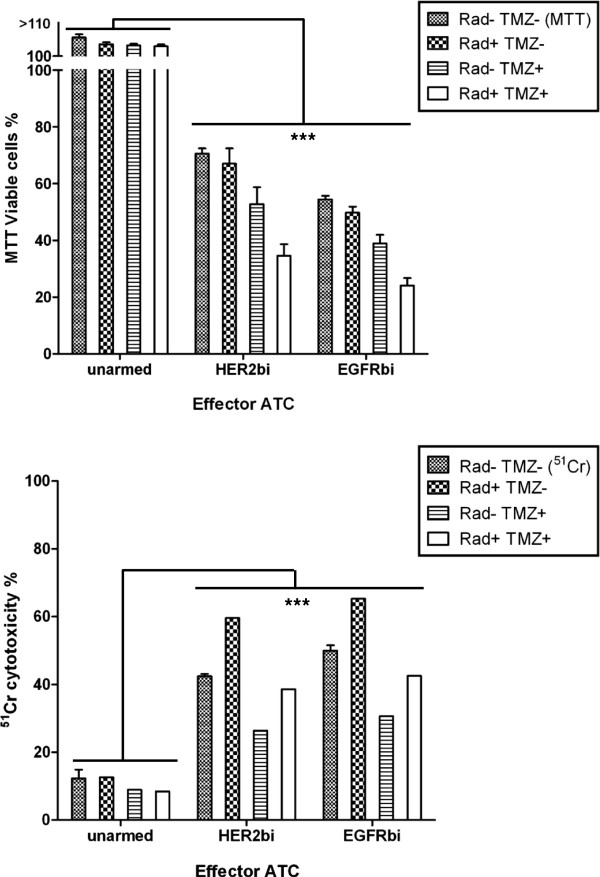
**Killing by armed ATC is resistant to both radiation and temozolomide. **TMZ-resistant U251MG cells were used as target cells for overnight killing by unarmed, HER2Bi-, and EGFRBi-armed ATC. The upper panel shows the MTT data and the lower panel shows ^51^Cr release data. Each panel has three sets of 4 columns. The E:T was 10:1. The individual sets represent the effects of unarmed, HER2Bi-armed, and EGFRBi-armed ATC, respectively. Within each data set, the 4 columns show the viability or cytotoxicity, respectively, for type of treatment with and without irradiation or TMZ (final 100 μM). The four sets of effector cells were untreated (Radiation– TMZ–), irradiated only (Radiation+ TMZ–), exposed to TMZ only (100 μM in the assay medium) (Radiation– TMZ+) and both irradiated and exposed to TMZ (Radiation+ TMZ+). Two assay plates were set up in parallel. The MTT data are shown as mean (± SEM) residual percent viable cells and the mean (± SEM) ^51^Cr release data as percent cytotoxicity. Two-way ANOVA of the MTT data show highly significant effects of both treatment and arming (p < 0.0001 for each), but no effect of interaction (p = 0.27). For the ^51^Cr data, treatment (p < 0.0001), arming (p < 0.0001) and the interaction of the two (p = 0.0003) are all highly significant.

Since TMZ and radiation are generally given after surgery for patients with glioblastoma, we determined whether armed ATC could kill glioma cells under comparable *in vitro* conditions. Our preliminary experiments show that the TMZ-resistant U251MG could be killed by armed ATC in the absence of TMZ (unpublished data) and other studies showing radioresistance of armed ATC effector function [14,15], we tested whether irradiation and TMZ would inhibit cytotoxicity mediated by unarmed and both HER2Bi- and EGFRBi-armed ATC. These studies were done in parallel using MTT and ^51^Cr release assays (Figure 6) and data were analyzed using non-parametric statistics.

In the MTT assay, unarmed ATC did not kill glioma targets. The ^51^Cr release cytotoxicity assay show <20% cytotoxicity for unarmed ATC alone and ATC that receive radiation alone, TMZ alone, and the combination of radiation and TMZ. Radiation alone had no effect on the viability of either HER2Bi- and EGFRBi-armed ATC (MTT data) but may have increased cytotoxicity (^51^Cr release data) although was not statistically significant (p=0.07). Exposure to TMZ alone showed a 25-28% reduction in MTT-viable cells with 38% reduction in ^51^Cr specific cytotoxicity. These data suggest that TMZ may be potentially toxic to ATC. Finally, combination of radiation and TMZ shows 50-55% reduction in viable cells, but only 9-15% reduction in cytotoxicity. Irradiation of armed ATC results in 34-38% reduction in MTT-viable cells when TMZ is present, but increased specific cytotoxicity of 38-46% (p=0.025). Therefore, the cytotoxic capacity of armed ATC is not only radioresistant but can kill in the presence of TMZ.

### Do glioma cells exhibit suppressive or contact-dependent inhibitory effects on ATC?

**Figure 7:**
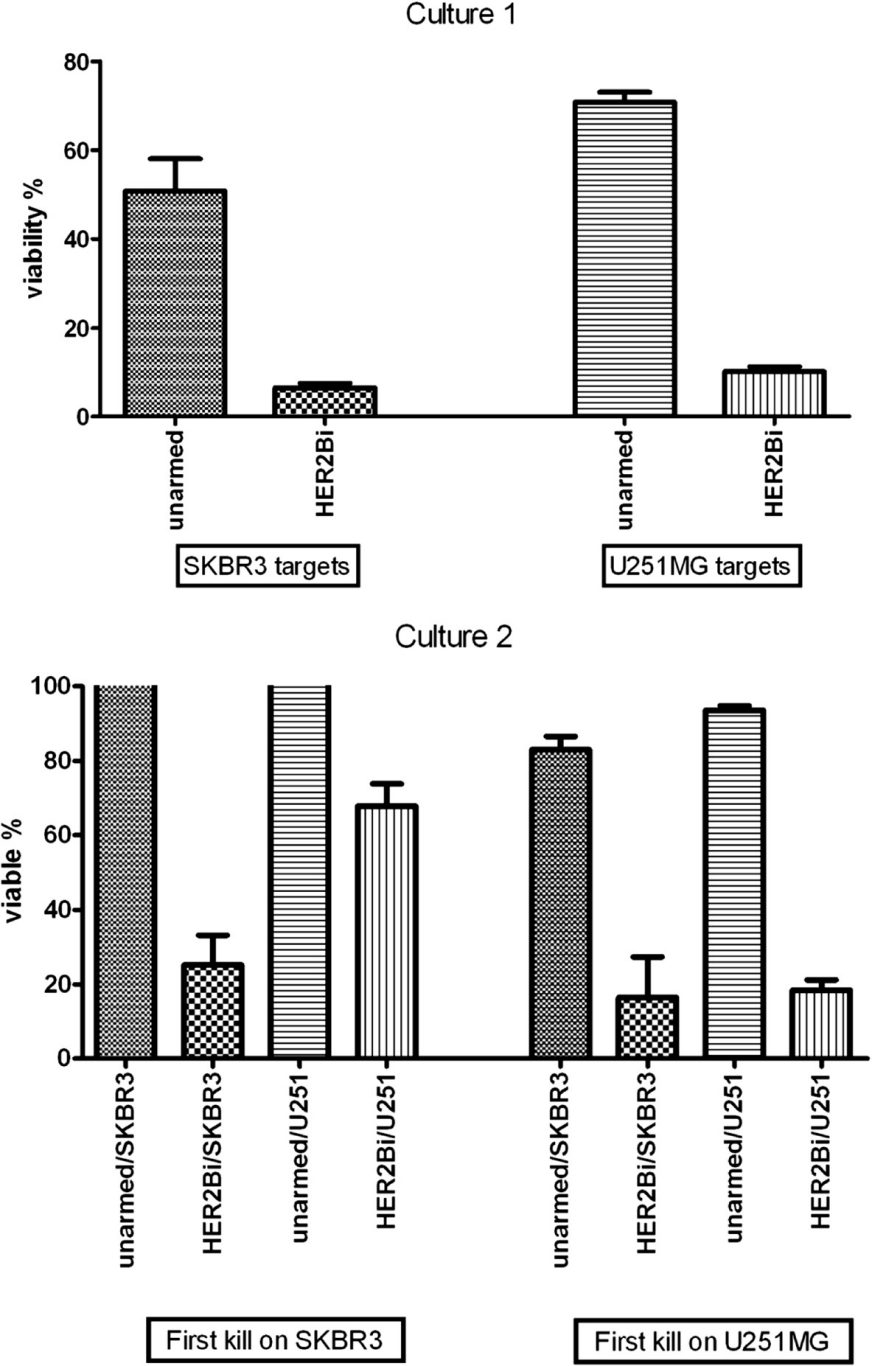
**Armed ATC show sequential killing and effector function is unaffected by contact with glioma cells. **In the first overnight culture (Culture 1) unarmed ATC or HER2Bi-armed ATC were incubated with SKBR3 or U251MG target cells (first kill) (E:T = 10:1). The effector cells were removed and MTT added to the wells to determine residual viability compared to control target cells to which no effectors had been added. Data are means (± SEM) of 9–11 cultures. After overnight incubation, without any additional IL-2, the unarmed or HER2Bi-armed ATC were used in a second culture (Culture 2), in which they were added to fresh targets or irrelevant targets (E:T = 10:1). After overnight incubation (second kill), the effectors were removed and MTT added to the wells to determine residual viability of the targets. Data are shown as mean (± SEM) of 4–7 cultures in each group.

To test whether glioma cells or normal astrocytes secrete molecules that are capable of inhibiting the function of cytotoxic T cells, we incubated ATC for 6 days in conditioned medium from both normal astrocytes and two separate *ex vivo* glioma lines. We then tested unarmed or armed ATC from these co-cultures for their ability to kill U118MG and U251MG target cells. Exposure of ATC or armed ATC to conditioned medium from normal astrocytes or gliomas did not inhibit their cytotoxic activity towards U118MG or U251MG target cells compared to the ability of control effectors that were not treated with conditioned medium (data not shown). Next, we asked whether cell-cell contact between armed ATC and target cells inhibited cytotoxicity. For this, we carried out a repeat killing assay, in a target cross-over experiment (Figure 7). First, unarmed and HER2Bi-armed ATC were incubated overnight, at E:T 10:1 with either SK-BR-3 (irrelevant human breast cancer cells) or U251MG cells. After removal of the non-adherent ATC, the viability via MTT was <10% (>90% killing) for HER2Bi on SK-BR-3 and U251MG targets (Culture 1 in Figure 7). The recovered armed and unarmed ATC from each co-culture were then re-plated onto cultures containing the same or other tumor cell lines to determine whether they would kill again and whether they would retain their specificity (Culture 2 in Figure 7). Specifically-armed ATC from SK-BR-3 co-culture showed reduced killing of U251MG cells and slight reduction in second killing of SK-BR-3 cells but ATC from U251 co-culture showed no reduction in killing for either cell lines. These data confirm that armed ATC can mediate their effects more than once; furthermore, co-cultures with gliomas cells did not suppress the ability of armed ATC to kill glioma cells.

### Are cytokines secreted by armed ATC?

**Figure 8:**
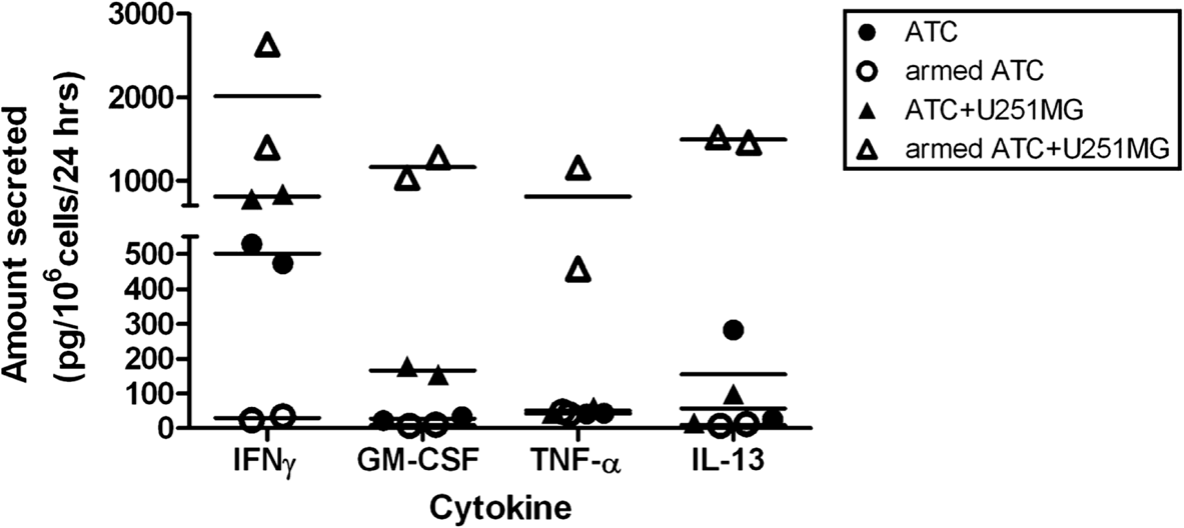
**Both Th1 and Th2 cytokines are secreted by armed ATC. **Unarmed or EGFRBi-armed ATC (10^7^) were incubated overnight alone or with U251MG cells (E:T = 25:1). The values are reported as pg/10^6^/24 hours in 1 ml cultures. The culture supernatant was assayed by Bio-Plex assay for the presence of Th1 and Th2 cytokines. Data shown are for each of the ATC donors individually; the horizontal line indicates the median value. The figure shows the concentrations only for those cytokines which showed differences between effectors alone and effectors plus targets.

Using ATC from two donors, we incubated unarmed and EGFRBi armed ATC both alone and with U251 cells (E:T = 25:1) and recovered the culture supernatants after overnight incubation. Cytokine levels in the culture supernatants were determined using the Bio-Plex assay. The data for cytokines that showed significant changes are shown in Figure 8 and reflect the means of the two donors. Similar cytokine profile was obtained with U87 and U118 cell lines (data not shown).

 Three Th1 cytokines (IFN-γ, GM-CSF, and TNF-α) and one Th2 cytokine (IL-13) showed increases in concentration when the armed ATC were incubated with targets cells. The unarmed ATC secreted 500—840 pg IFN-γ/10^6^cells/24 hrs when incubated alone or with target cells, however armed ATC incubated alone secreted little or no IFN-γ, whereas incubation EGFRBi armed ATC with U251 cells elicited induced 1400—2600 pg/ml of IFN-γ secretion. The other cytokines in Figure 8 show very low baseline concentrations that are clearly increased when they are co-incubated with target cells.

## Discussion

High-grade gliomas are aggressive tumors and respond poorly to all treatment modalities. Since these tumors are highly infiltrative, surgical resection leaves a residuum of cells responsible for recurrence. Malignant gliomas are almost uniformly lethal, with a median survival of about 15 months. This underscores the need for effective, non-toxic strategies that can eliminate the residual tumor cells and also, perhaps, immunize the endogenous immune system against the tumor.

 Our immunotherapy approach originates from a series of studies, in which T cells have been redirected to EpCam on adenocarcinomas [3]; HER2/*neu* on prostate [7], breast [6], and ovarian cancer [5]; EGFR on a variety of tumor types [4]; and CD20 on malignant B cells [7]. These studies were performed *in vitro*, in small animal models, and have progressed to clinical trials [16]. In a recent study, we have shown the involvement of Granzyme B (GrzB) and IFN-γ signaling pathways in BiAb armed ATC mediated cytotoxicity of target cells [17].

This study shows that both long-term glioma lines and primary cultures of freshly-resected glioblastoma are efficiently killed by ATC armed with either HER2Bi or EGFRBi, indicating that both antigens are potential targets. We also showed that killing is not enhanced by using both BiAbs simultaneously. It may be that, in future studies, individual gliomas will show differential expression of HER2/*neu* and EGFR and that it will be prudent to both phenotype and functionally test each tumor as a target, in order to choose the best BiAb.

Cells with the stem-like property of self-renewal and the ability to differentiate into the bulk population of tumor cells have been identified in a number of different solid tumor types, amongst the best characterized of which are breast [18] and gliomas [19]. CD133 was the initial marker identified as characterizing the glioma cancer stem cell, although there are subsequent reports of CD133– cells with similar behavior [20]. The important additional features of stem cells are that they have been postulated to be both chemo- and radioresistant and to be responsible for the extensive infiltration seen in gliomas [21]. The ability to kill CD133+ and CD133− cells, as shown above, indicates that these stem-like cells may be susceptible to killing in this system.

We showed that TMZ-resistant U251MG cells are also susceptible to targeted killing and that armed ATC still kill in the presence of TMZ. TMZ-resistant U251MG cells were <1% CD133+. This may be due to the length of time this line has been in culture, but their susceptibility does indicate that glioma cells that do not have stem-like properties but acquire chemoresistance are also suitable targets for BiAb-armed ATC. We also demonstrated the radioresistance of armed ATC effector function and an indication that irradiation of ATC may cause an increase in cytotoxicity. One possible interpretation is that there is a radiosensitive population of cells in the ATC that suppresses cytotoxic activity. Whether this involves active suppression or merely reflects death of the radiosensitive cells remains unclear. These results suggest that patients undergoing conventional chemoradiation may be suitable candidates for treatment with armed ATCs.

Some tumor types have been shown to produce soluble factors that inhibit immune effectors [22] and others to express membrane molecules, such as FasL that actually kill effectors [23-29]. We ruled-out the former by showing that long-term incubation of ATC in culture supernatants from immortalized malignant glioma lines and *ex vivo* gliomas and non-neoplastic astrocytes do not inhibit killing activity. We tested the latter, using long-term tumor lines and showed that these, at least, do not diminish the cytotoxic activity of armed ATC. That is, co-culture of armed ATC with glioma cells permits repeat killing [10].

 Finally, we also showed that when armed ATC are incubated with targets, there is increased secretion of three Th1 cytokines (IFN-γ, GM-CSF, and TNF-α) and one Th2 cytokine (IL-13). The armed ATC are the presumed source of the Th1 cytokines and their secretion would serve to activate microglia (IFN-γ), act as an adjuvant for immunization of endogenous lymphocytes (GM-CSF) and potentially augment the killing of target tumor cells (TNF-α). The cellular origin of the IL-13 is probably also the ATC, since CD8+ T cells have been shown to secrete this cytokine [30]. However, an IL-13 receptor is expressed on some glioma cells [31] and IL-13 has been shown to be an autocrine growth factor for both Reed-Sternberg cells in Hodgkin’s disease [32-35] and pancreatic cancer [36]. The potential contribution of the glioma cells to the increased IL-13 is under investigation.

Several studies have shown promising results in glioblastoma using various immunotherapeutic approaches [37]. Lymphokine-activated killer (LAK) cells generated from PBMC by co-culture with IL-2 have been reported to selectively kill glioma cells *in vitro* and when placed into the resection cavity with minimal systemic or neurological side effects [38,39]. In the first study in which cells were targeted to WHO grade III/IV gliomas, LAK cells were treated with a conjugate of anti-CD3 cross-linked to the NE-150 monoclonal antibody which recognizes an epitope of NCAM [40]. In the control group which received untreated LAK cells, 9/10 patients experienced recurrence within 1 year and 8/10 died within 4 years. In the experimental group, 2/10 showed no response, 4/10 showed regression and 4/10 had complete response. No recurrences occurred during 10—18 months of follow-up in the 8 patients showing partial or complete response. Subsequently, others have reported the use of different BiAbs both *in vitro* and early-stage clinical trials [41-46].

## Conclusions

In conclusion, high-grade gliomas appear to be suitable targets for specific targeting by armed ATC. Should these results be confirmed in animal studies, comparable to those performed with other tumor targets, this approach may represent an additional adjuvant treatment for this devastating malignancy.

## Competing interests

LGL is founder of Transtarget, Inc. All other authors report no conflict of interest.

## Authors’ contributions

IMZ, AT, and ON acquired the data and performed the statistical analysis. IMZ, GRB, LGL, and SM designed the study. All authors interpreted the data and helped draft the manuscript. AT, LGL, and SM revised the manuscript. All authors read and approved the final manuscript.

## Pre-publication history

The pre-publication history for this paper can be accessed here:

http://www.biomedcentral.com/1471-2407/13/83/prepub
